# Second Annual Meeting of the New Jersey State Dental Society

**Published:** 1871-12

**Authors:** 


### Proceedings of Societies.

### SECOND ANNUAL MEETING OF THE NEW JERSEY STATE DENTAL SOCIETY.

The second annual meeting of the Society was called to order by the President.

After the reading of the minutes, and a favorable report of the Executive Committee, seventeen names were added to the roll of the Society. Dr. Stockton then addressed the meeting.

He commenced by giving an epitome of the history of dentistry from the time of the Pharaohs to the present hour, and congratulated his hearers upon the progress and present status of dentistry as a profession. He spoke upon what he conceived to be the important elements of successful practice. It was the fashion with a certain class of minds to belittle success, but the more judicious in this age hold to the opinion that men who make their way to the front and maintain a high position in any legitimate pursuit in life must have merit. To prostrate one's self before the shrine of riches and power might be called "flunkeyism," but reverential respect for success in any honorable pursuit is worthy of worship. Occasionally pretenders meet with success, and people speak of luck; but true success comes not by chance, for men apparently adapt the same means to the same end; one fails, the other succeeds, and we say the latter is the more fortunate. But in reality their means were different. It is not by doing the right thing, but by doing it in the right way, at the right time, that we achieve the greatest triumphs in life. Again, we must measure a man's success, not by the relation which his achievements bear to those of others, but by what he has himself accomplished. If he has attained the object he had in view—provided it be worthy an honorable ambition—he is a successful man.

Concentration of thought and effort upon one's specific pursuit is an important element of success. Self reliance is a valuable quality, but the aid of professional brethren is by no means to be condemned. The demand now is for associated effort. No practitioner could make his mark who ignored it. We can often learn more at the dinner table, from an associate, than in our own laboratory studying a difficult case. Successful men have not confined themselves to direct action, or looked only to immediate results. Not a few failures result from a lack of faith in and fellowship with professional brethren. The longer we live, the more we will be convinced that social intercourse is a material means to success. Through its instrumentality valuable information is imparted and received, improvements in mechanical appliances, aids, &c., suggested, and experiences in difficult operations communicated. Frequently the gain is palpable at once, and we count the advantage as we take off our dress coat; if not, it will make itself manifest sooner or later.

Keeping up with the profession involves hard labor, but we should not be content with that. All acting upon that principle, our profession would soon come to a stand. Contentment with what is already known would of necessity end all progression. We are to be imparters as well as receivers—to endeavor always to add something to the department of knowledge to which our calling relates. Attainment to a full knowledge of the science and principles of his profession, as far as practiced, is far from a despicable object of ambition; but to enlarge its usefulness and augment its achievements is certainly more worthy of one's aspirations.

Over-sensitiveness oftentimes interferes with a man's success. We must not be over-sensitive to attacks upon our pride. Every successful man has had much to mortify him in the career; he has borne many rebuffs, sustained many failures. Nor should we be discouraged over our mistakes and failures. Scarcely a practitioner among us but will admit he was (in a measure) made by his failures, and that what he once thought his hard fate was in reality his good fortune. Error, properly improved, becomes the stepping stone to the highest success. Many a promising practitioner has been permanently injured by early success. But it will not profit us, or our reputation, or the profession, to publish our mistakes. Be it understood that by over-sensitiveness is not meant over-scrupulousness. Be very scrupulous. Never get up any pretentious humbug; never seek to undervalue the work of another; never advertise cheaper or better work than your brother to draw away patients.

In estimating the sources of success, account must be taken of our habits and temperaments. Men otherwise well qualified have failed to make a name in the profession from neglect of attention to these important particulars. Most of us are sensible, at intervals, of feebleness and weariness, when we are seemingly incapable of any exertion; and we hesitate at such times, to know what to do—whether it were better to make the will assert itself, and go on in spite of weakness and lassitude and continued entreaties from the frail flesh, or let nature have her own way and succumb at once. Again, there is fear of doing what we have in hand badly, perhaps of having it all to do over, or of making on the minds of those we wish to influence favorably an impression of weakness. On the other hand, there is danger of making compromises with our active powers and yielding to the temptations of indolence. We are in danger of mistaking idleness for debility.

It is a difficult thing to lay down specific rules. Each practitioner should study his own temperament. If we know we are not indolent—if we have for the most part pleasure in the practice of our profession, and never need the spur—we may safely pause when our energies are flagging.

The speaker then alluded briefly to the question of manner as involving an important element of success, though it was impossible to lay down specific rules upon the subject. He spoke in favor of liberality in extending the benefits of improvements and discoveries from one member of the profession to another, deprecating anxiety for patent rights, and pleaded for encouragement and aid to unfortunate struggling practitioners. He closed by referring to the principles of Divine revelation as those upon which all true success is founded.

Drs. Maynard, of Elizabeth, Fitch, of New York, and Armes, were elected honorary members.

The election of officers resulted as follows:

*President*—A. W. Kingsley, of Elizabeth.

*Vice President*—G. F. J. Colburn, of Newark.

*Treasurer*—Lewis Reading, of Trenton.

*Secretary*—E. F. Hanks, of Rahway.

*Executive Committee*—William Dibble, Elizabeth; Thos. Stephens, Trenton; J. C. Robbins, Jersey City; C. S. Stockton, Mt. Holly; Leo H DeLange, Bordentown.

It was resolved that a committee of three be appointed to take into consideration the regulation of the practice of dentistry in the State of New Jersey, and, if practicable, have a law passed that no one hereafter shall be allowed to commence the practice of dentistry unless they shall have passed a satisfactory examination before a board appointed in accordance with the act, graduates of dental colleges excepted.

Drs. Stockton, Reading and Colburn were appointed on that committee.

After due consideration, Long Branch was selected as the next place of meeting.

Dr. G. F. J. Colburn, of Newark, was selected to deliver the next annual address.

Some discussion then ensued on "Mechanical Dentistry."

Dr. Kingsley condemned Weston's metal in toto.

Straight's flexible edge met with no better success at the hands of Dr. Chew.

Dr. J. C. Robbins objected very strongly to the odor of the celluloid base.

Dr. Hayhurs thought the camphor in the plate would be liable to become dissolved in the mouth.

SECOND DAY.

Drs. DeLange and Hanks gave a clinic filling teeth with gold, both using the rubber dam. Dr. DeLange demonstrated his method of capping the nerve, using oxychloride of zinc, finishing the rather difficult operation of filling the posterior proximal surface of an upper molar tooth without previous separation or preparation very creditably. Dr. Hanks inserted a simple crown filling in a lower molar.

Dr. Colburn does not use the rubber dam, because he gets along perfectly well without it.

Dr. Stevens thinks it a valuable discovery; had used it since last Society meeting with gratifying results.

Dr. Hanks cured a case of alveolar abscess that pointed on the chin by injecting the aromatic tincture of sulphuric acid; was indebted to Dr. Atkinson for the mode of treatment.

Dr. Kingsley gave a case in practice. Necrosis followed abscess; dissected the gum and took away the sockets almost entirely on one side of the maxillary.

Dr. Colburn described Bacon's method of entrapping dentists who use the rubber without license.

On motion, it was resolved that the New Jersey State Dental Society strongly protest against and denounce the practice of advertising cheap dentistry now in vogue by those claiming to be dentists.

The Society then adjourned.

